# Supplementary material for: Anti-Metastatic Effect of Pyruvate Dehydrogenase Kinase 4 Inhibition in Bladder Cancer via the ERK, SRC, and JNK Pathways
Source: Int J Mol Sci. 2022 Oct 31;23(21):13240. doi: 10.3390/ijms232113240 (PMC9658024; doi:10.3390/ijms232113240)
Supplement: Supplementary file 1 [file ijms-23-13240-s001.zip › ijms-1943994-supplementary.pdf]

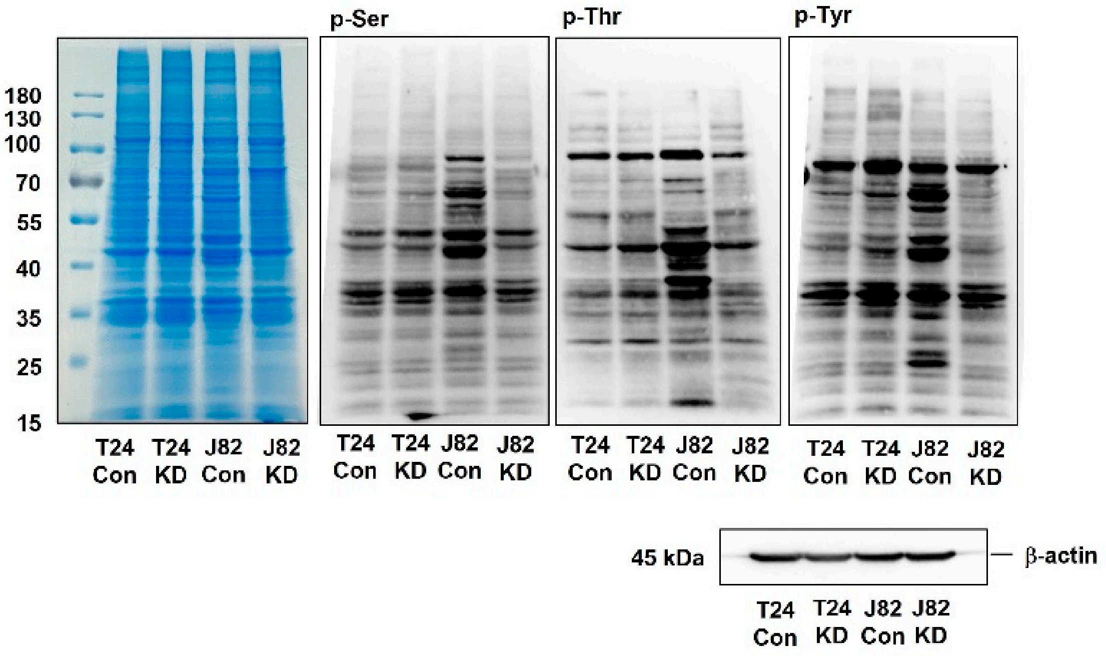


**Figure S1.** Screening of protein phosphorylation (S/T/Y) profiling in bladder cancer cell lines. Western blotting scanning in bladder cancer cells of phosphorylation that occurred on serine, threonine, and tyrosine. KD, knockdown; Control, wild-type.

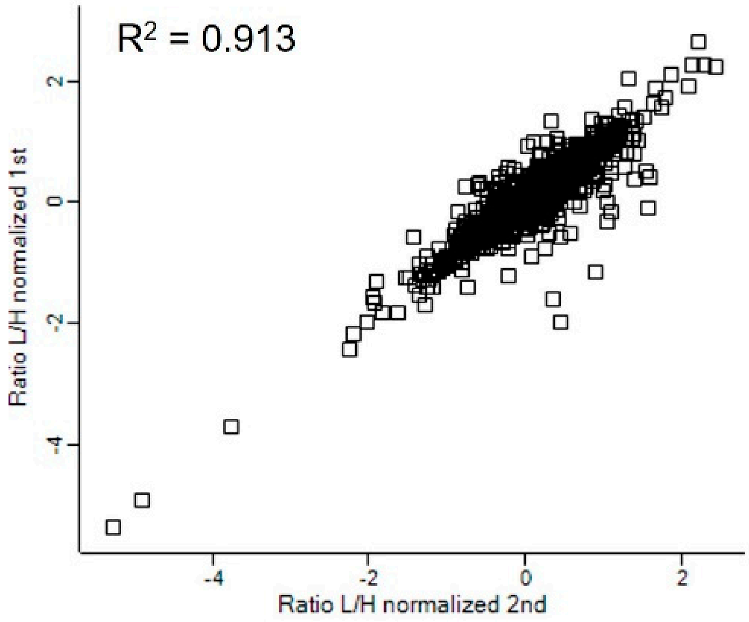


**Figure S2.** Phosphoproteomics correlation of bladder cancer. Pearson correlation of phosphoproteomes between technical samples.

**Table S1.** Summary of differentially expressed proteins.

| UniProt IDs | Protein Names | Gene Names |
|---|---|---|
| F8WF12 | Golgin subfamily B member 1 | GOLGB1 |
| P42677 | 40S ribosomal protein S27 (Metallopan-stimulin 1) (MPS-1) (Small ribosomal subunit protein eS27) | RPS27 |
| H7C5M1 | RNA-binding protein 34 (Fragment) | RBM34 |

| | | |
|---|---|---|
| P10412 | Histone H1.4 (Histone H1b) (Histone H1s-4) | H1-4 |
| F2Z2T1 | Nuclear cap-binding protein subunit 1 | NCBP1 |
| B1AMJ5 | CDK5 regulatory subunit-associated protein 2 (Fragment) | CDK5RAP2 |
| K7EN42 | Glycylpeptide N-tetradecanoyltransferase 1 (Fragment) | NMT1 |
| D6RI08 | OCIA domain-containing protein 1 (Fragment) | OCIAD1 |
| Q53EU6 | Glycerol-3-phosphate acyltransferase 3 (GPAT-3) (EC 2.3.1.15) (1-acyl-sn-glycerol-3-phosphate O-acyltransferase 10) (AGPAT 10) (1-acyl-sn-glycerol-3-phosphate O-acyltransferase 9) (1-AGP acyltransferase 9) (1-AGPAT 9) (EC 2.3.1.51) (Acyl-CoA:glycerol-3-phosphate acyltransferase 3) (hGPAT3) (Lung cancer metastasis-associated protein 1) (Lysophosphatidic acid acyltransferase theta) (LPAAT-theta) (MAG-1) | GPAT3 |
| C9JEN3 | Protein lifeguard 3 (Fragment) | TMBIM1 |
| F8WBL2 | Bystin | BYSL |
| Q96RS0 | Trimethylguanosine synthase (EC 2.1.1.-) (CLL-associated antigen KW-2) (Cap-specific guanine-N2 methyltransferase) (Hepatocellular carcinoma-associated antigen 137) (Nuclear receptor coactivator 6-interacting protein) (PRIP-interacting protein with methyltransferase motif) (PIMT) (PIPMT) | TGS1 |
| J3KP30 | Deoxynucleotidyltransferase terminal-interacting protein 2 | DNTTIP2 |
| O75683 | Surfeit locus protein 6 | SURF6 |
| O95297 | Myelin protein zero-like protein 1 (Protein zero-related) | MPZL1 |
| K7EPZ6 | Methyl-CpG-binding domain protein 1 (Fragment) | MBD1 |
| G5E9C0 | SP110 nuclear body protein, isoform CRA_b (Sp110 nuclear body protein) | SP110 |
| P07550 | Beta-2 adrenergic receptor (Beta-2 adrenoreceptor) (Beta-2 adrenoceptor) | ADRB2 |
| F8WBW8 | BAH and coiled-coil domain-containing protein 1 | BAHCC1 |
| Q8NEF9 | Serum response factor-binding protein 1 (SRF-dependent transcription regulation-associated protein) (p49/STRAP) | SRFBP1 |
| E7ES96 | Presenilin (EC 3.4.23.-) | PSEN1 |
| P28715 | DNA repair protein complementing XP-G cells (EC 3.1.-.-) (DNA excision repair protein ERCC-5) (Xeroderma pigmentosum group G-complementing protein) | ERCC5 |
| F5H6E2 | Unconventional myosin-Ic | MYO1C |
| Q8TB61 | Adenosine 3′-phospho 5′-phosphosulfate transporter 1 (PAPS transporter 1) (Putative MAPK-activating protein PM15) (Putative NF-kappa-B-activating protein 48) (Solute carrier family 35 member B2) | SLC35B2 |
| O75151 | Lysine-specific demethylase PHF2 (EC 1.14.11.-) (GRC5) (PHD finger protein 2) | PHF2 |
| Q13206 | Probable ATP-dependent RNA helicase DDX10 (EC 3.6.4.13) (DEAD box protein 10) | DDX10 |
| O95453 | Poly(A)-specific ribonuclease PARN (EC 3.1.13.4) (Deadenylating nuclease) (Deadenylation nuclease) (Polyadenylate-specific ribonuclease) | PARN |
| Q9ULF5 | Zinc transporter ZIP10 (Solute carrier family 39 member 10) (Zrt- and Irt-like protein 10) (ZIP-10) | SLC39A10 |
| B4DTR1 | Receptor tyrosine-protein kinase erbB-2 (cDNA FLJ59426, highly similar to Receptor tyrosine-protein kinase erbB-2) | ERBB2 |
| Q13206 | Probable ATP-dependent RNA helicase DDX10 (EC 3.6.4.13) (DEAD box protein 10) | DDX10 |
| Q13428 | Treacle protein (Treacher Collins syndrome protein) | TCOF1 |
| E9PFD7 | Receptor protein-tyrosine kinase (EC 2.7.10.1) | EGFR |
| Q92508 | Piezo-type mechanosensitive ion channel component 1 (Membrane protein induced by beta-amyloid treatment) (Mib) (Protein FAM38A) | PIEZO1 |
| Q92508 | Piezo-type mechanosensitive ion channel component 1 (Membrane protein induced by beta-amyloid treatment) (Mib) (Protein FAM38A) | PIEZO1 |
| O00161 | Synaptosomal-associated protein 23 (SNAP-23) (Vesicle-membrane fusion protein SNAP-23) | SNAP23 |
| O00161 | Synaptosomal-associated protein 23 (SNAP-23) (Vesicle-membrane fusion protein SNAP-23) | SNAP23 |
| J3QSV6 | Ribosomal L1 domain-containing protein 1 (Fragment) | RSL1D1 |
| P46821 | Microtubule-associated protein 1B (MAP-1B) [Cleaved into: MAP1B heavy chain; MAP1 light chain LC1] | MAP1B |
| J3QSV6 | Ribosomal L1 domain-containing protein 1 (Fragment) | RSL1D1 |
| O00161 | Synaptosomal-associated protein 23 (SNAP-23) (Vesicle-membrane fusion protein SNAP-23) | SNAP23 |

| | | |
|---|---|---|
| O43707 | Alpha-actinin-4 (Non-muscle alpha-actinin 4) | ACTN4 |
| Q9H3N1 | Thioredoxin-related transmembrane protein 1 (Thioredoxin domain-containing protein 1) (Transmembrane Trx-related protein) | TMX1 |
| Q9BQG0 | Myb-binding protein 1A | MYBBP1A |
| Q9H6F5 | Coiled-coil domain-containing protein 86 (Cytokine-induced protein with coiled-coil domain) | CCDC86 |
| A0A0G2JNZ2 | Protein scribble homolog | SCRIB |
| A0A0G2JNZ2 | Protein scribble homolog | SCRIB |
| A0A2U3TZL8 | Kinesin-like protein | KIF23 |
| P07355 | Annexin A2 (Annexin II) (Annexin-2) (Calpactin I heavy chain) (Calpactin-1 heavy chain) (Chromobindin-8) (Lipocortin II) (Placental anticoagulant protein IV) (PAP-IV) (Protein I) (p36) | ANXA2 |
| P07355 | Annexin A2 (Annexin II) (Annexin-2) (Calpactin I heavy chain) (Calpactin-1 heavy chain) (Chromobindin-8) (Lipocortin II) (Placental anticoagulant protein IV) (PAP-IV) (Protein I) (p36) | ANXA2 |
| Q96PD2 | Discoidin, CUB and LCCL domain-containing protein 2 (CUB, LCCL and coagulation factor V/VIII-homology domains protein 1) (Endothelial and smooth muscle cell-derived neuropilin-like protein) | DCBLD2 |
| P20337 | Ras-related protein Rab-3B | RAB3B |
| Q9BVJ6 | U3 small nucleolar RNA-associated protein 14 homolog A (Antigen NY-CO-16) (Serologically defined colon cancer antigen 16) | UTP14A |
| Q9BQG0 | Myb-binding protein 1A | MYBBP1A |
| Q6ZRP7 | Sulfhydryl oxidase 2 (EC 1.8.3.2) (Neuroblastoma-derived sulfhydryl oxidase) (Quiescin Q6-like protein 1) | QSOX2 |
| Q8IWS0 | PHD finger protein 6 (PHD-like zinc finger protein) | PHF6 |
| P46013 | Proliferation marker protein Ki-67 (Antigen identified by monoclonal antibody Ki-67) (Antigen KI-67) (Antigen Ki67) | MKI67 |
| Q13501 | Sequestosome-1 (EBI3-associated protein of 60 kDa) (EBIAP) (p60) (Phosphotyrosine-independent ligand for the Lck SH2 domain of 62 kDa) (Ubiquitin-binding protein p62) | SQSTM1 |
| Q8WYP5 | Protein ELYS (Embryonic large molecule derived from yolk sac) (Protein MEL-28) (Putative AT-hook-containing transcription factor 1) | AHCTF1 |
| Q9NR30 | Nucleolar RNA helicase 2 (EC 3.6.4.13) (DEAD box protein 21) (Gu-alpha) (Nucleolar RNA helicase Gu) (Nucleolar RNA helicase II) (RH II/Gu) | DDX21 |
| P02545 | Prelamin-A/C [Cleaved into: Lamin-A/C (70 kDa lamin) (Renal carcinoma antigen NY-REN-32)] | LMNA |
| Q8N556 | Actin filament-associated protein 1 (110 kDa actin filament-associated protein) (AFAP-110) | AFAP1 |
| Q8NFH5 | Nucleoporin NUP35 (35 kDa nucleoporin) (Mitotic phosphoprotein 44) (MP-44) (Nuclear pore complex protein Nup53) (Nucleoporin NUP53) | NUP35 |
| P49006 | MARCKS-related protein (MARCKS-like protein 1) (Macrophage myristoylated alanine-rich C kinase substrate) (Mac-MARCKS) (MacMARCKS) | MARCKSL1 |
| P11388 | DNA topoisomerase 2-alpha (EC 5.6.2.2) (DNA topoisomerase II, alpha isozyme) | TOP2A |
| P02545 | Prelamin-A/C [Cleaved into: Lamin-A/C (70 kDa lamin) (Renal carcinoma antigen NY-REN-32)] | LMNA |
| P20700 | Lamin-B1 | LMNB1 |
| Q09666 | Neuroblast differentiation-associated protein AHNAK (Desmoyokin) | AHNAK |
| P46087 | Probable 28S rRNA (cytosine(4447)-C(5))-methyltransferase (EC 2.1.1.-) (Nucleolar protein 1) (Nucleolar protein 2 homolog) (Proliferating-cell nucleolar antigen p120) (Proliferation-associated nucleolar protein p120) | NOP2 |
| Q9UQ35 | Serine/arginine repetitive matrix protein 2 (300 kDa nuclear matrix antigen) (Serine/arginine-rich splicing factor-related nuclear matrix protein of 300 kDa) (SR-related nuclear matrix protein of 300 kDa) (Ser/Arg-related nuclear matrix protein of 300 kDa) (Splicing coactivator subunit SRm300) (Tax-responsive enhancer element-binding protein 803) (TaxREB803) | SRRM2 |
| Q09666 | Neuroblast differentiation-associated protein AHNAK (Desmoyokin) | AHNAK |
| Q9P2E9 | Ribosome-binding protein 1 (180 kDa ribosome receptor homolog) (RRp) (ES/130-related protein) (Ribosome receptor protein) | RRBP1 |
| Q09666 | Neuroblast differentiation-associated protein AHNAK (Desmoyokin) | AHNAK |

| | | |
|---|---|---|
| F8W6I7 | Helix-destabilizing protein (Heterogeneous nuclear ribonucleoprotein A1) (Heterogeneous nuclear ribonucleoprotein A1, N-terminally processed) (hnRNP core protein A1) | HNRNPA1 |
| Q9H6F5 | Coiled-coil domain-containing protein 86 (Cytokine-induced protein with coiled-coil domain) | CCDC86 |
| P07355 | Annexin A2 (Annexin II) (Annexin-2) (Calpactin I heavy chain) (Calpactin-1 heavy chain) (Chromobindin-8) (Lipocortin II) (Placental anticoagulant protein IV) (PAP-IV) (Protein I) (p36) | ANXA2 |
| O94804 | Serine/threonine-protein kinase 10 (EC 2.7.11.1) (Lymphocyte-oriented kinase) | STK10 |
| A0A087WWP4 | RNA-binding protein 15 | RBM15 |
| A0A087WWP4 | RNA-binding protein 15 | RBM15 |
| P16401 | Histone H1.5 (Histone H1a) (Histone H1b) (Histone H1s-3) | H1-5 |

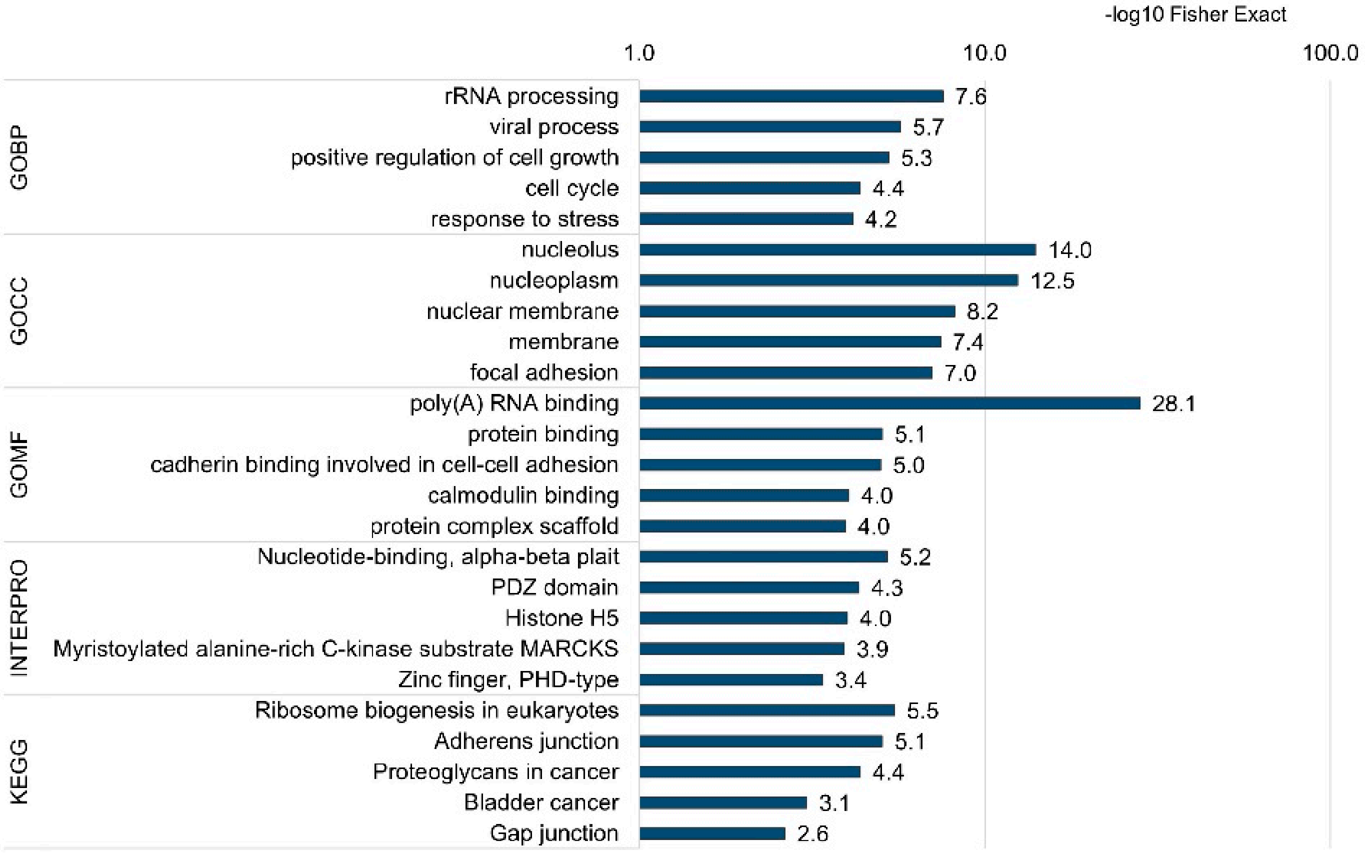


**Figure S3.** DAVID analysis of differentially expressed proteins. Downregulated phosphoproteins. GOBP, Gene Ontology Biological Process; CC: cellular component; MF, molecular function; KEGG, Kyoto Encyclopedia of Genes and Genomes.
